# Antimicrobial Resistance Determinants Circulating among Thermophilic *Campylobacter* Isolates Recovered from Broilers in Ireland Over a One-Year Period

**DOI:** 10.3390/antibiotics9060308

**Published:** 2020-06-08

**Authors:** Caoimhe T. Lynch, Helen Lynch, Sarah Burke, Kayleigh Hawkins, Colin Buttimer, Conor Mc Carthy, John Egan, Paul Whyte, Declan Bolton, Aidan Coffey, Brigid Lucey

**Affiliations:** 1 Department of Biological Sciences, Cork Institute of Technology, Bishopstown, T12 P928 Cork, Ireland; caoimhe.lynch@mycit.ie (C.T.L.); sburke1@mycit.ie (S.B.); kayleigh.hawkins@mycit.ie (K.H.); conor.mccarthy6@mycit.ie (C.M.C.); aidan.coffey@cit.ie (A.C.); 2 NRL Campylobacter, Backweston Laboratory Complex, Young’s Cross, Celbridge, W23 X3PH Kildare, Ireland; helen.lynch@agriculture.gov.ie (H.L.); john.egan@ucd.ie (J.E.); 3 School of Veterinary Medicine, University College Dublin, Belfield, D04 V1W8 Dublin 4, Ireland; paul.whyte@ucd.ie; 4 APC Microbiome Ireland, University College Cork, T12 YT20 Cork, Ireland; colin.buttimer@ucc.ie; 5 Teagasc Food Research Centre, Ashtown, D15 DY05 Dublin 15, Ireland; declan.bolton@teagasc.ie

**Keywords:** *Campylobacter*, antimicrobial resistance, ciprofloxacin, tetracycline, aminoglycosides, macrolides

## Abstract

Campylobacteriosis is the leading cause of human bacterial gastroenteritis, very often associated with poultry consumption. Thermophilic *Campylobacter* (*Campylobacter jejuni* and *Campylobacter coli*) isolates (*n* = 158) recovered from broiler neck skin and caecal contents in Ireland over a one-year period, resistant to at least one of three clinically relevant antimicrobial classes, were screened for resistance determinants. All ciprofloxacin-resistant isolates (*n* = 99) harboured the C257T nucleotide mutation (conferring the Thr-86-Ile substitution) in conjunction with other synonymous and nonsynonymous mutations, which may have epidemiological value. The A2075G nucleotide mutation and amino acid substitutions in L4 and L22 were detected in all erythromycin-resistant isolates (*n* = 5). The *tetO* gene was detected in 100% (*n* = 119) of tetracycline-resistant isolates and three of which were found to harbour the mosaic tetracycline resistance gene *tetO/32/O*. Two streptomycin-resistant *C. jejuni* isolates (isolated from the same flock) harboured *ant(6)-Ib*, located in a multidrug resistance genomic island, containing aminoglycoside, streptothricin (*satA*) and tetracycline resistance genes (truncated *tetO* and mosaic *tetO/32/O*). The *ant(6)-Ie* gene was identified in two streptomycin-resistant *C. coli* isolates. This study highlights the widespread acquisition of antimicrobial resistance determinants among chicken-associated *Campylobacter* isolates, through horizontal gene transfer or clonal expansion of resistant lineages. The stability of such resistance determinants is compounded by the fluidity of mobile genetic element.

## 1. Introduction

*Campylobacter* is the most commonly reported foodborne bacterial pathogen causing human gastroenteritis in the European Union (EU) and Ireland, most often associated with the broiler reservoir [1]. Ireland was found to have a 98% prevalence of campylobacter-contaminated broiler carcasses in 2008 [2]. Frequent isolation of antimicrobial-resistant *Campylobacter* spp. of food animal origin continues to limit the spectrum of clinically useful antimicrobials and is internationally recognised as a major societal challenge. Veterinary antimicrobials used therapeutically and prophylactically are often the same as, or belong to the same class as those used clinically [3].

Macrolides, fluoroquinolones and aminoglycosides are classified as critically important antimicrobials, while tetracycline is considered a highly important antimicrobial [3]. Resistance to (fluoro)quinolones and tetracyclines is highly prevalent in clinical and broiler-associated *Campylobacter* spp. isolates, while resistance to erythromycin is typically low to moderate across Europe [4,5,6]. Macrolides are the first line antibiotic for the treatment of enteric gastroenteritis, while fluoroquinolones and tetracyclines remain as alternatives [7,8,9]. Systemic infections are routinely treated with aminoglycosides [9,10] and resistance to aminoglycosides is low in clinical and broiler isolates across Europe [5]. Cross-resistance between aminoglycoside antimicrobials is incomplete and although streptomycin is not used clinically to treat campylobacteriosis, resistance can be used as an indicator for acquired aminoglycoside resistance genes. 

In Gram negative bacteria, DNA gyrase is the primary target of fluoroquinolones [11]. DNA gyrase is a heterotetrameric type IIA topoisomerase, consisting of two polypeptide subunits (GyrA and GyrB, encoded by *gyrA* and *gyrB*, respectively), catalysing ATP-dependent negative supercoiling of DNA to regulate replication, repair and gene expression [12,13,14]. Resistance to (fluoro)quinolones among *Campylobacter* spp. is largely mediated by chromosomal mutations in the quinolone resistance-determining region (QRDR) of *gyrA*, typically conferred by the C257T nucleotide mutation (Thr-86-Ile) [15]. The QRDR is located near the Tyr-125 active site, involved in DNA-protein bridge formation during DNA strand passage [15]. 

Macrolides act by binding to the 50S bacterial ribosomal subunit and inhibit translational elongation, and interfere with protein synthesis and subsequent ribosomal subunit assembly [16,17]. Polymorphisms in the 23S ribosomal RNA (rRNA), mutations in 50S ribosomal proteins L4 and L22 (encoded by *rplD* and *rplV*, respectively) or the presence of the emerging *ermB* gene contribute to macrolide resistance [8,18]. Βeta-hairpin extensions from 50S ribosomal proteins L4 and L22 are involved in the regulation of nascent peptide exit from the large ribosomal subunit [19,20]. Mutations in L4 and L22, combined with the overexpression of antimicrobial efflux genes have been reported to contribute to high-level macrolide resistance [21]. The ribosomal methylase encoded by *ermB* was reported recently for the first time in thermophilic *Campylobacter* spp., located on a chromosomal multidrug resistance genomic island (MDRGI) (likely originating from a Gram positive species) in a high-level erythromycin-resistant *Campylobacter coli* (*C. coli*) isolate (ZTC113) of swine origin in China [8,22]. ErmB dimethylates adenine at position 2074 of the 23S rRNA gene, reducing the binding affinity of macrolides [23]. 

The intrinsic, chromosomally encoded resistance-nodulation-division (RND) CmeABC (Campylobacter multidrug efflux) efflux pump in *Campylobacter* spp. contributes to baseline resistance against structurally diverse antimicrobials [6,24,25,26]. The *cmeABC* operon encodes a tripartite multidrug efflux pump that consists of an outer membrane channel protein (*cmeC*), an inner membrane efflux transporter (*cmeB*) and a periplasmic fusion protein (*cmeA*) [27]. Repressor (*cmeR*) binding to an inverted repeat (IR) (TGTAATAAATATTACA) in the intergenic region between *cmeR* and *cmeA* transcriptionally represses the *cmeABC* operon [28,29]. Consequently, polymorphisms in the repressing site induce efflux pump overexpression and enhanced resistance to antimicrobials, most notably, erythromycin [8,30].

Tetracycline resistance in *Campylobacter* spp. is largely conferred by a ribosomal protection protein (RPP), TetO, capable of displacing tetracycline from its primary binding site on the 30S ribosomal subunit [17,31]. Bacterial resistance to tetracycline is also associated with ATP-dependent efflux or enzymatic inactivation of tetracycline [32,33,34]. *Campylobacter tetO* can be located chromosomally but is often plasmid-mediated [31,33,35,36,37,38,39]. Tetracycline RPPs are widely distributed among bacterial genera and it has been reported that the *tetO* gene exists in at least eleven bacterial genera, including four Gram negative and seven Gram positive genera [33]. The conserved acquisition of *tetO* between members of different bacterial genera indicates that conjugative plasmids, transposons, or recombination events contribute to the dissemination and maintenance of the *tetO* gene [37]. 

Although *tetO* acquisition is the most prevalent genetic event conferring tetracycline resistance among *Campylobacter* spp., mosaic *tet* genes (specifically *tetO/32/O*) have also been reported within the genus [34]. Mosaic tetracycline resistance genes are derived from the interclass (double-crossover) recombination of two or more RPP-encoding gene (predominantly *tetO*, *W*, *32* [34,40,41,42] and *tetM*, *S* [43]) to form functional chimera [34]. Mosaic *tet* genes are widespread among Gram positive and Gram negative genera in human and animal isolates [41].

Aminoglycosides are broad spectrum antimicrobials and inhibit protein synthesis by binding to 16S rRNA of the 30S ribosome [44,45]. *Campylobacter* spp. resistance to aminoglycosides is mediated by reduced antimicrobial binding affinity for target sites due to enzymatic modification, via acetylation, phosphorylation or adenylation of amino or hydroxyl groups of the aminocyclitol nucleus or sugar moieties [44,45,46]. Although there are two main nomenclature systems used to identify aminoglycoside modifying enzymes [47,48,49], we followed the system proposed by Shaw et al. (1993), later extended to include an expanded panel of aminoglycoside 6-nucleotidyltransferases (also known as adenyltransferases) [50,51]. The designation proposed by Shaw et al. (1993) is as follows: the type of modification (nucleotidyltransferase/adenyltransferases (ANT)); the modification site (6’); a roman numeral to denote unique resistance profiles (I), and a letter to represent unique protein sequences (b) [48]. Genes for ANT enzymes are found on transposons, plasmids or chromosome, often in associated with other resistance genes and very often as part of the transposon-associated aminoglycoside-streptothricin resistance gene cluster (*ant(6)-I-sat4-aphA3*), first isolated from *Staphylococci* [52,53]. ANT(6)-I encoding genes are widely distributed among clinical and animal streptomycin-resistant thermophilic *Campylobacter* spp. isolates [51].

Despite the high prevalence of *Campylobacter* in broilers on the island of Ireland, in the last twenty years, only a few reports of the molecular mechanisms contributing to resistance exist for broiler [54,55,56,57,58,59], clinical [54,56,59,60,61], domestic animal [62] or ruminant [56,63] isolates. We report the antimicrobial resistance determinants circulating among 158 resistant *Campylobacter jejuni* (*C. jejuni*) and *C. coli* isolates recovered from Irish broiler neck skin and caecal samples over a one-year period (2017–2018).

## 2. Results

### 2.1. Fluoroquinolone Resistance

Isolates resistant to ciprofloxacin/nalidixic acid were screened for mutations in the *gyrA* gene and 100% of isolates (*n* = 99) harboured a C257T point mutation, which is the dominant mutation conferring resistance among campylobacters. Resistant isolates were grouped into *C. jejuni gyrA* and *C. coli gyrA* (arbitrarily named GTJs and GTCs, respectively) sequence types based on the presence of synonymous and nonsynonymous mutations present in the portion of the *gyrA* gene sequenced (Table 1). Ciprofloxacin-resistant *C. jejuni* isolates (*n* = 85) were grouped into three GTJs (GTJ-I, -II, and -III). A large proportion (47.1%) carried the Thr-86-Ile substitution exclusively (GTJ1). Synonymous mutations T72C, C243T, T357C, C360T, C471T, T483C, and C622T were exclusively associated with *C. jejuni* and were present in both GTJ-II and GTJ-III. Nonsynonymous Ser-22-Gly (A64G) and Ala-206-Thr (G616A) mutations were present in 35.3% and 17.7% of isolates of ciprofloxacin-resistant *C. jejuni* isolates, respectively and were the basis of defining GTJ-II and GTJ-III, respectively. Both GTJ-II and GTJ-III were associated with the Asn-203-Ser (A608G) substitution. All CIP-resistant *C. coli* isolates tested (*n* = 14) harboured one nonsynonymous mutation only (Thr-86-Ile), but were grouped into seven GTCs based on the presence of various synonymous mutations (Table 1).

No high-level moxifloxacin resistance was detected among the 99 (fluoro)quinolone-resistant isolates tested and minimum inhibitory concentrations (MICs) ranged from 0.5–8 mg/L.

### 2.2. Erythromycin Resistance

Five erythromycin-resistant isolates were screened for mutations contributing to erythromycin resistance. All five erythromycin-resistant isolates harboured the A2075G mutation in the 23S rRNA gene. A T82C mutation (Ser-28-Pro substitution) in the *rplD* gene was detected in the five isolates, and the partial sequences shared 100% homology with the *rplD* gene of erythromycin-sensitive *C. coli* isolates (GenBank accession numbers: MH084640.1 and MH084639.1) [64]. Identical mutations were also observed in the *rplV* sequence in all erythromycin-resistant isolates: double point mutation at positions 308 and 309 (Ala-103-Val), A325G (Thr-109-Ala), a double point mutation at positions 332 and 333 (Ala-111-Glu), G340A (Ala-114-Thr) and C358A (Pro-120-Thr). Nonsynonymous mutations (T282A, C294T, A306G, T321G) in *rplV* were also identified in all erythromycin-resistant isolates. Partial *rplV* sequences in this study were homologous to a high-level erythromycin-resistant clinical *C. coli* isolate *rplV* gene (GenBank accession number: GU384982.1) [28]. Similarly, all five erythromycin-resistant isolates harboured a 9 base pair deletion (positions 45–54) and an insertion at position 45 (G) in *cmeR*-*cmeA* intergenic region, upstream of the IR (positions 66-80), homologous to sequences derived from erythromycin-resistant (GenBank accession number: FJ797673.1) and erythromycin-sensitive (GenBank accession number: FJ797671.1) strains [24]. The *ermB* gene was not detected among the erythromycin-resistant isolates tested.

### 2.3. Tetracycline Resistance

**Figure 1:**
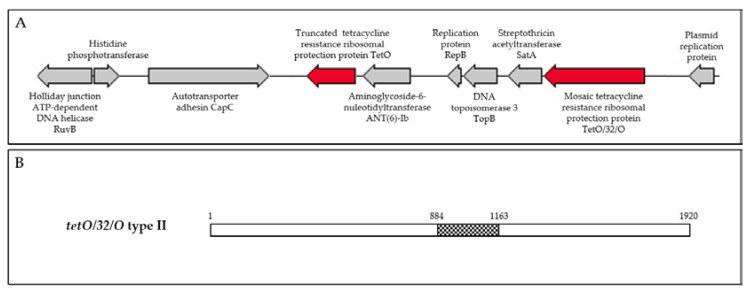
(**A**) Schematic of *Campylobacter jejuni* isolate CITCj625-18 multidrug resistance genomic island. (**B**) Schematic of the mosaic *tetO/32/O* type II gene detected in CITCj625-18 and CITCj727-18. Figure adapted from Warburton et al. (2016). White bars are *tetO* and central, checked bar is *tet32* (297 bp).

A portion of the *tetO* gene was detected in 100% of tetracycline-resistant isolates (*n* = 119). Three isolates (CITCj625-18, CITCj727-18, and CITCc3448-18), accounting for 2.5% of the tetracycline-resistant isolates, harboured the mosaic *tetO/32/O* type II gene, confirmed by PCR/partial sequencing and genomic sequencing (Figure 1B). Isolates CITCj625-18 and CITCj727-18 carried identical mosaic tetracycline genes, but differed from CITCCc3448-18 by Thr-118-Ile and Glu-176-Asp substitutions and A684G and A789G point mutations. The mosaic tetracycline gene detected among the Irish broilers isolates in this study was very similar to a *Streptococcus suis* (*S. suis*) (GenBank accession number: KY994102.1) *tetO/32/O* gene (Table 2). Equally, the TetO/32/O sequences were >99.5% identical to *Campylobacter* spp. (GenBank accession numbers: WP_052855148.1) and *Clostridiales* (WP_117823345.1) TetO/32/O sequences. 

### 2.4. Streptomycin Resistance

*C. jejuni* isolates CITCj625-18 and CITCj727-18 were found to harbour multiple resistance genes, including a truncated *tetO* (873 bp, truncated at the 3’ end), mosaic *tetO/32/O* type II (1920 bp), aminoglycoside-6-nucleotidyltransferase (*ant(6)-Ib*)), and streptothricin acetyltransferase (*satA*). These antimicrobial resistance genes were located circumjacent to proteins involved in replication and recombination (Table 3, Figure 1A). The MDRGI contained 10 open reading frames in a region of 35.9% GC content, compared to the average genomic GC content of 30.5%. Genomic sequences of *C. jejuni* isolates CITCj625-18 (first thin) (1,630,363 bp) and CITCj727-18 (final thin, isolated a week later) (1,636,524 bp) were nearly identical with an average nucleotide identity (ANI) of 99.99% and 30.5% GC content, indicating that the isolates had been circulating within and had been maintained by the flock. The pair belonged to sequence type ST-45 clonal complex (ST-137) and harboured LOS locus class C.

CITCc1631-18 and CITCc3448-18 belonged to ST-828 clonal complex (ST-6543 and ST-1096, respectively) and harboured the *ant(6)-Ie* gene (900 bp), with almost identical sequences (99.89% identity). ANT(6)-Ie in this study shared 99.66% amino acid identity with each other, where CITCc3448-18 harboured a nonsynonymous G820A mutation (Val-274-Ile) and was identical to a *C. coli* ANT(6)-Ie protein (GenBank: WP_052786298.1). CITCc1631-18 and CITCc3448-18 ANT(6)-Ie shared between 29.7-36.1% identity to ANT(6)-Ia, ANT(6)-Ib, ANT(6)-Ic, and ANT(6)-Id amino acid sequences (GenBank: AFJ97257.1, AFJ97264.1, AAR10415.1, and WP_001258597.1, respectively) [50,51,65]. 

Aminoglycoside 3-*N*-acetyltransferase (AAC(3)) (261 amino acids) was also detected in both CITCj625-18 and CITCj727-18, 44.3% identical to a aminoglycoside 3-N-acetyltransferase (AAC(3)) variant (239 amino acids) in both CITCc1631-18 and CITCc3448-18. However, these isolates were gentamicin-susceptible, although this protein may confer resistance to other aminoglycoside antibiotics [66]. The *C. coli* variant was identical to AAC(3) genes reported in *Campylobacter* spp., 37.5% identical to AAC(3)-Ia described in *Serratia marcescens* (GenBank accession number: Q7B9H0), and 19–32.25% similar to AAC(3) orthologous, including types AAC(3)-Ib/Ic/Id/Ie/IIa/IIb/IIc/IId/IIe/IIf/IIIa/IIIb/IIIc/IVa/VIa/VIIIa/Ixa/Xa/XIa. The AAC(3) variant in CITCj625-18 and CITCj727-18 was identical to *Campylobacter* AAC(3) variants and was 19.3–25.84% similar to orthologues reported in other bacterial genera.

### 2.5. Overall Distribution of Antimicrobial Resistance

**Figure 2:**
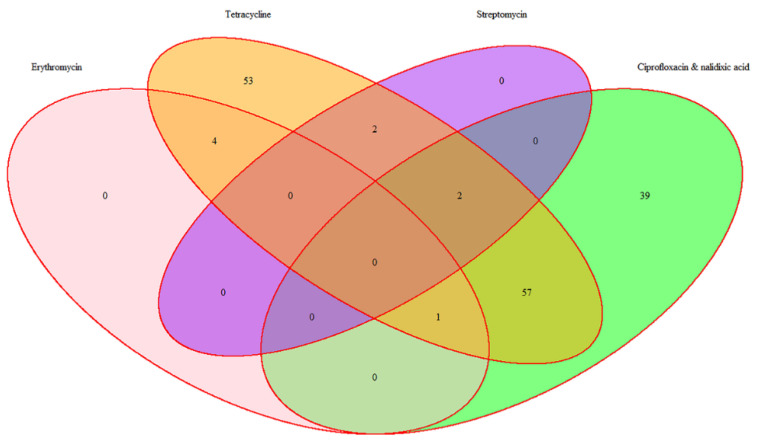
Distribution of antimicrobial resistance among 158 thermophilic *Campylobacter* spp.

The antimicrobial resistance rates of this pool of broiler-associated thermophilic *Campylobacter* spp. isolates (resistant to at least one antimicrobial; n = 158) have been detailed previously [67], and are summarised below (Figure 2).

## 3. Discussion

This study reports the antimicrobial resistance determinants circulating among Irish broiler-associated *Campylobacter* isolates, collected throughout the Republic of Ireland, from the three largest poultry processors, over a one-year period (2017–2018). 

The Thr-86-Ile mutation is the predominant genetic alteration conferring (fluoro)quinolone resistance among *Campylobacter* spp. [68], and was detected in all (fluoro)quinolone-resistant isolates (*n* = 99) tested in the current study, similar to reports published worldwide [6,69,70,71]. Some studies have not detected the Thr-86-Ile mutation universally in the QRDR of (fluoro)quinolone-resistant *Campylobacter* spp. isolates [58,62,72], indicating that other factors are responsible for, or contribute to, (fluoro)quinolone resistance.

Fluoroquinolone-resistant *Campylobacter* spp. are ecologically competitive and persistent even in the absence of antimicrobial selective pressure [73,74,75]. Despite a distinct rise in ciprofloxacin resistance among broiler *Campylobacter* isolates (3.1% to 28.9%) in Ireland between 1998 and 2000 [54,76], in 2017–8 resistance to ciprofloxacin remained stable (28.3%) [67]. Clonal expansion of resistant lineages has likely contributed to the persistence of ciprofloxacin resistance in Ireland, considering that fluoroquinolones typically account for less than 1% of all veterinary antimicrobials sold in Ireland [77,78,79].

*C. jejuni* isolates harbouring multiple amino acid substitutions in the *gyrA* QRDR (GTJ-II and GTJ-III) (Table 1) had ciprofloxacin MICs ranging from 8–16 mg/L, except one GTJ-II isolate had an MIC of 4 mg/L, while isolates harbouring the Thr-86-Ile mutation exclusively (GTJ-I) had MICs ranging from 4–16 mg/L. Similarly, Ekkapobyotin et al. (2008) observed varying ciprofloxacin/nalidixic acid MICs in isolates harbouring identical GyrA amino acid substitutions [80]. Moreover, the Ser-22-Gly, Asn-203-Ser and Ala-206-Thr mutations have been reported in fluoroquinolone-sensitive strains [81,82,83]. To confirm the apparent lack of involvement of these accessory *gyrA* mutations in the development of fluoroquinolone resistance, the introduction of these mutations in fluoroquinolone-susceptible strains could be investigated. Authors have previously reported that double mutations in *gyrA* (at amino acid positions 86 and 90) were necessary to produce high level moxifloxacin resistance [84,85]. Moxifloxacin is a potent fluoroquinolone with activity against fluoroquinolone-resistant campylobacters that harbour a single mutation in *gyrA* [86]. In this study, no high-level resistance to moxifloxacin was observed among the (fluoro)quinolone-resistant isolates. These data indicate that mutations outside the *gyrA* QRDR have a negligible effect on (fluoro)quinolone resistance.

Variation in *gyrA* alleles within a population of *Campylobacter* isolates have been identified as epidemiological markers and may serve as a supplementary approach to classical epidemiological typing methods [81,83,87,88]. In our study, the GTs detected were species specific, although Ragimbeau et al. (2014) reported the presence of a typical *C. coli gyrA* type in 0.23% (*n* = 1) of 430 *C. jejuni* isolates tested, and 1.4% (*n* = 4) *C. coli* isolates harboured a typical *C. jejuni gyrA* type. The amino acid substitutions present in each of the three GTJ (Table 1) lineages have been associated with poultry *Campylobacter* isolates previously [89,90,91]. Only one nonsynonymous mutation (Thr-86-Ile) was detected among the seven GTCs detected (Table 1), similar to previous studies reporting a single nonsynonymous mutation (Thr-86-Ile substitution) present in (fluoro)quinolone-resistant *C. coli* QRDR sequences [83,89,90]. It is likely that additional variants of *Campylobacter* spp. *gyrA* alleles exist, and may reflect ecological evolution [83]. 

Identical mutations in the 23S rRNA, *rplD*, and *rplV* genes were detected in erythromycin-resistant isolates (*n* = 5), while MICs ranged from 128 mg/L to ≥ 128 mg/L. Three of these isolates were collected from the same flock in north-central Ireland while one isolate was collected from a farm approximately 10 km away, the following week. The fifth erythromycin-resistant isolate was recovered from a farm in the mid-south-west of Ireland, two months previously, but all birds from these farms were processed in the same processing plant. 

The A2075G point mutation in the 23S rRNA gene remains the most prevalent genetic event conferring macrolide resistance [8,92] and was detected in all erythromycin-resistant isolates in this study. The 23S rRNA A2074G and A2074C mutations were not detected. Mutations in the *rplV* gene, encoding the 50S ribosomal protein L22 were detected in the C-terminal region (amino acids 109–142) [18], including Thr-109-Ala, Ala-111-Glu, Ala-114-Thr, and Pro-120-Thr. Nonsynonymous mutations (T282A and C294T) were also observed in the region encoding the highly conserved β-hairpin loop at amino acids 78–98 [18]. Mutations in the RplD β-hairpin (spanning amino acid positions 55–77 [18,93]) are often associated with bacterial macrolide resistance, and such mutations were not observed among the Irish erythromycin-resistant isolates tested in this study. Moreover, polymorphisms were detected outside the *cmeR* regulatory IR. The effects of mutations detected in this study in *rplD*, *rplV*, and the intergenic region of *cmeR*-*cmeA* are unknown, but may contribute to erythromycin resistance.

The *ermB* gene was not detected among the erythromycin-resistant isolates tested. Resistance mediated by *ermB* in *Campylobacter* spp. is largely confined to China, which may reflect the extensive use of antimicrobials in food producing animals in China [8]. Three reports of genetically distinct *ermB*-positive *C. coli* isolates recovered from poultry exist in Europe [94,95,96] and an *ermB*-positive isolate was detected for the first time in the United States in a *C. jejuni* isolate of clinical origin [97], while the *ermB* gene was recently detected in 18.3% of 240 thermophilic *Campylobacter* spp. retail meat associated-isolates in South Africa [98]. Mutations in *C. jejuni* 23S rRNA has been associated with a fitness cost and reduced doubling times [99,100,101], although tolerance to low temperatures may facilitate persistence in the environment and transmission of resistant strains through the food supply [100]. 

All tetracycline-resistant isolates harboured a portion of the *tetO* gene, while three isolates harboured the mosaic *tetO/32/O* type II gene were detected. Mosaic tetracycline resistance genes in *Campylobacter* spp. are typically derived from *tetO* and *tet32* sequences in the type II conformation, with a shorter central *tet32* segment [34], although there are limited reports of these resistance genes circulating among *Campylobacter* spp. The first mosaic tetracycline RPP gene was detected in *Megasphera elsdenii*, composed of a central *tetW* region flanked by two *tetO* regions [102]. However, the progenitors of these mosaic genes are based only on the order in which they were discovered, and the current classification system does not adequately reflect the variable nature of tetracycline RPPs [34,43]. The *tetO* primers [103] used in this study amplify a region at the beginning of the *tetO* gene and enable the detection of mosaic *tet* genes with a central portion flanked by an initial *tetO* region until position 228. The *tetO/32/O* type II gene reported here was associated exclusively with an MIC of 64 µg/L. However, 27 of 119 (22.7%) tetracycline-resistant *Campylobacter* spp. isolates tested had MICs of 64 µg/L or ≥ 64 µg/L, indicating that other factors contribute to enhanced tetracycline resistance. It should be noted that in this study, all three isolates harbouring mosaic tetracycline genes were also co-resistant to streptomycin, enabling co-selection and persistence of these antimicrobials. The burden of mosaic tetracycline resistance genes within the genus should be considered as part of the approach to elucidate developing and newly acquired antimicrobial resistance determinants within the genus. 

All streptomycin resistant (*n* = 4) isolates had MICs of ≥ 16 mg/L. Streptomycin-resistant *C. jejuni* isolates CITCj625-18 and CITCj727-18 (ST-137) harboured sialylated LOS locus class C, which has been identified as a risk factor for post-infectious Guillain-Barré syndrome and increased severity of enteric disease [104]. CITCj625 and CITCj727-18 belonged to ST-137 and members of the diverse *C. jejuni* ST-45 clonal complex [105,106]. ST-137 is frequently isolated from cases of enteric campylobacteriosis [105] and broilers/avian [107,108,109], porcine [105], and bovine [110] hosts. The ST-137 genotype is widely dispersed and represents an ecologically successful clone [106]. A study by Dearlove et al. (2016) reported that ST-45 clonal complex was a generalist lineage capable of frequent transmission between hosts. 

Genomic sequencing of tetracycline-/streptomycin-resistant *C. jejuni* isolates (CITCj625-18 and CITCj727-18, isolated from the same flock, first and final thin) revealed identical genes in a multidrug resistance genomic island (Table 3, Figure 1A). Both isolates harboured a truncated *tetO* gene and a mosaic *tetO/32/O* type II gene, homologous that of Gram positive (GenBank accession number: KY994102.1) and *Campylobacter* spp. (GenBank accession number: WP_002823161.1). The presence of multiple *tet* genes (coding for similar or different mechanisms) in Gram negative isolates has also been documented [33]. However, the truncated form detected in this study (CITCj625-18 and CITCj727-18) is likely a remnant of a region of insertion or recombination. Truncated *tetO* genes have also been reported in *C. coli* MDGRI containing *aadE* (*ant(6)-Ib*) and *ermB* [94,111]. Aminoglycoside (*ant(6)-Ib* (867 bp)) and streptothricin resistance (*satA*) genes were also located within the multidrug resistance island (Figure 1A). Streptothricin acetyltransferase A (*satA*) is frequently reported in Gram positive bacilli [112] and shares less than 40% identity with the streptothricin acetyltransferase A (*sat4*) reported in *Campylobacter* [52,111,113]. Plasmid replication proteins within the MDRGI suggest a plasmid as the insertion vehicle of the resistance genes.

CITCc3448-18 belonged to ST-828 clonal complex (ST-1096). ST-1096 has been isolated from a case of gastroenteritis in the United Kingdom (UK) in 2016 [114] and has also been previously reported in *C. coli* of swine origin in Spain, America, and Grenada [51,115,116]. CITCc1631 was ST-6543 (ST-828 clonal complex), which has been associated with clinical and chicken-associated isolates [117]. There are a total of fifteen depositions (all are UK associated) of *C. coli* ST-6543 on the PubMLST *Campylobacter* database at the time of writing [114], including eleven clinical isolates (faeces), two chicken-associated isolates, and two isolates with no source allocation. 

Both streptomycin-resistant *C. coli* isolates (CITCc1631-18 and CITCc3448-18) harboured the emerging *ant(6)-Ie* gene (900 bp), found widely disseminated among clinical and animal *C. coli* isolates [51,65]. Unlike other *Campylobacter ant(6)* genes, *ant(6)-Ie* appears to be inherent to *C. coli* and does not have a Gram positive ancestor [65]. The *ant(6)-Ie* gene was originally detected in a hypervariable genomic region, unaccompanied by other resistance genes [65]. Similarly, the CITCc1631-18 and CITCc3448-18 *ant(6)-Ie* genes were located chromosomally, and were not located near other resistance determinants. Both streptomycin-resistant *C. coli* isolates were co-resistant to ciprofloxacin/nalidixic acid (GTC-V) and tetracycline. *C. coli* isolate 1631-18 harboured *tetO* (1920 bp), while *C. coli* isolate CITCc3448-18 also harboured the mosaic tetracycline resistance gene, *tetO/32/O* (1290 bp), highly homologous with that detected in CITCj625-18 and CITCj727-18. 

## 4. Materials and Methods 

### 4.1. Bacterial Isolate Culture Conditions and Susceptibility Testing 

A total of 350 thermophilic *Campylobacter* isolates (296 *C. jejuni* and 54 *C. coli*) were recovered from free range and intensively-reared broiler carcasses (neck skin and caecal contents) using ISO 10272-2:2017 [2]. Isolates were collected between September 2017 and September 2018, from the three largest poultry processing plants in the Republic of Ireland. The collection of isolates was speciated using matrix-assisted laser desorption ionisation-time of flight (MALDI-TOF) mass spectrometry (MS) (Bruker, Billerica, MA, United States). Isolates were previously tested [67] for their MIC to six clinically relevant antimicrobials, namely ciprofloxacin, nalidixic acid, erythromycin, tetracycline, gentamicin, and streptomycin according to ISO 20776:2006 and EC Decision 2013/652/EU [118,119]. Overall, 158 (140 *C. jejuni* and 18 *C. coli*) isolates tested were resistant to at least one antimicrobial and were subsequently tested for resistance determinants.

### 4.2. DNA Extraction

Briefly, isolates were recovered from −80 °C on Columbia blood agar (Fannin Ltd, Dublin, Ireland) and incubated for 24 h at 42 °C, under microaerobic conditions (5% O_2_, 10% CO_2_, 85% N_2_) and subcultured. DNA was extracted using the PureLink Genomic DNA Mini Kit (Invitrogen, Carlsbad, CA, USA), according to manufacturer’s instructions and DNA was standardised to 50–100 ng/μL.

### 4.3. Genotypic Characterisation of Antimicrobial Resistance—PCR Amplification and Sequencing 

Primer sets, target genes and annealing temperatures are listed in Table 4. Primers to detect mosaic tetracycline resistance genes (*tetO/32/O* and *tetO/W/O*) were designed on SnapGene2.3.2 software (from Insightful Science; available at snapgene.com) and regions of primer complementarity were assessed on Primer-BLAST [120].

PCRs were performed with 2.5 U of Amplitaq polymerase (Applied Biosystems, Foster City, CA, USA), 1 × PCR buffer I and 2.5 mM magnesium chloride (Applied Biosystems), 0.2 mM of each deoxyribonucleotide (Sigma Aldrich, St Louis, MO, USA), 0.2 pmol/μL of each primer, and 1 µL (1–2 ng/μL) of DNA. Reaction conditions were denaturation at 94 °C for 2 min, 35 cycles of denaturation at 94 °C for 30 s, annealing (Table 4) for 30 s and extension at 72 °C for 1 min followed by a final extension at 72 °C for 5 min. PCR products were purified using the High Pure PCR Purification Kit (Sigma Aldrich). Purified PCR products were Sanger sequenced (forward and reverse reads) (Table 4) by Eurofins Genomics (Eurofins Genomics, Ebersberg, Germany). Consensus sequences were aligned and assembled on SeqMan Pro (Lasergene) (DNAStar, Madison, WI, USA).

Ciprofloxacin-resistant isolates (*n* = 99) were screened for mutations in the QRDR of the *gyrA* gene [15]. Products were purified and sequenced, as described above. Consensus sequences were aligned to the *gyrA* of the reference *C. jejuni* (GenBank accession number: L04566.1 and AL111168.1) and *C. coli* sequences (GenBank accession number: AF092101.1 and NZ_UIGM01000003.1) on SnapGene 2.3.2. 

The primers used for the amplification and sequencing of domain V of 23S rRNA, *rplD*, and *rplV* (encoding L4 and L22 ribosomal proteins, respectively) and the regulatory site of the *cmeABC* operon in five erythromycin-resistant *C. coli* isolates are listed in Table 4. Partial multiple alignment to reference *C. coli* type strain NCTC 11366 (ATCC 33559) 23S rRNA (GenBank accession number: GQ167698.1), *rplD* (GenBank accession number: DQ639752.1 and UIGM01000003.1), *rplV* (GenBank accession number: UIGM01000003.1) and *cmeABC* operon (GenBank accession number: FJ797670.1) sequences was performed on SnapGene 2.3.2. Isolates were also screened for the presence of *ermB*, according to Wang et al. (2014). 

Tetracycline-resistant isolates (*n* = 119) were screened for the presence of *tetO*, according to Aminov et al. (2001) and products were visualised on 2% agarose gel electrophoresis. The *tetO* amplicon of a tetracycline-resistant *C. jejuni* isolate (CITCj382-18) from this study was purified and sequenced (as described above) as a positive control. Consensus sequences were aligned to the *C. jejuni tetO* gene (GenBank accession number: M18896.2).

Primers were designed to target *tetO/W/O* (Table 4) based on alignments between *tetO* (GenBank accession number: M18896.2) and mosaic *tetO/WO* genes (GenBank accession numbers: EF065524.1 and AY196921.1). Two *tetO/32/O*-targeting primers (Table 4) were designed based on regions of homology between *tetO* (GenBank accession number: M18896.2) and *tetO/32/O* type I genes with a longer central *tet32* region (GenBank accession numbers: AJ295238.3 and JQ740052.1) and *tetO/32/O* type II genes with a shorter central *tet32* segment (GenBank accession numbers: KY994102.1, FP929050.1, AIOQ01000025.1, NZ_AUJS01000017.1 and AABYPB010000033.1) [34].

Streptomycin-resistant isolates (*n* = 4) were screened for sialylated LOS locus class A/B and C (Table 4) by PCR, using *C. jejuni* 81-176 (ATCC BAA2151) and *C. jejuni* NCTC 11168 (DSM 27585), respectively, as positive controls. 

### 4.4. Moxifloxacin Minimum Inhibitory Concentration Testing

All (fluoro)quinolone-resistant isolates (*n* = 99) were tested for moxifloxacin susceptibility. Briefly, isolates were recovered from −80 °C on CBA (Fannin Ltd) for 24 h at 42 °C under microaerobic conditions, and subcultured to CBA for 20 ± 2 h at 42 °C under microaerobic conditions. In microtiter plates, 100 µL serial dilutions of moxifloxacin (Sigma Aldrich) were prepared in of Mueller Hinton broth with lysed horse blood (Thermo Fisher Scientific, Waltham, MA, USA) ranging from 0.125–16 mg/L. A 0.5 McFarland inoculum was prepared in 5 mL demineralised water (Thermo Fisher Scientific) and 100 µL was transferred to 11 mL of Mueller Hinton broth with lysed horse blood (Thermo Fisher Scientific). Moxifloxacin serial dilutions were inoculated with 100 µL of cell suspension and incubated for 20 ± 2 hat 42 °C under microaerobic conditions.

### 4.5. Genome Sequencing and Genomic Analysis

The genomes of four streptomycin-resistant isolates (*C. jejuni* isolates CITCj625-18 and CITCj727-18 and *C. coli* isolates CITCc1631-18 and CITCc3448-18) were sequenced. DNA was quantified in triplicate with the Quant-iT dsDNA Assay Kit (Thermo Fisher Scientific). Genomic DNA libraries were prepared using the Nextera-XT protocol (Illumina, San Diego, CA, USA), with changes including 2 ng of input DNA and a minute PCR elongation time. DNA quantification and library preparation were performed on a Hamilton Microlab STAR system. Pooled libraries were quantified using the Kapa Biosystems library quantification kit on a Roche light cycler 96 qPCR machine. Libraries were sequenced on the Illumina HiSeq using a 250 bp paired-end protocol. Reads were adapter trimmed using Trimmomatic 0.30 with a sliding window quality cut-off of Q15 [124]. De novo assembly was performed using SPAdes version 3.7 [125] and assembly quality was assessed using QUAST [126].

Genomes were annotated using Prokka 1.14.3 [127]. Similarity searches were performed using the BLAST suit of programs [128] and InterProScan [129]. Multi locus sequence typing (MLST) patterns was determined using PubMLST [114]. ANI was calculated using EzBioCloud [130].

### 4.6. Data Availability 

This whole-genome project has been deposited at DDBJ/ENA/GenBank under the accession number PRJNA612628. CITCj625-18, CITCj727-18, CITCc1631-18, and CITCc3448-18 genomes can be accessed using SAMN14379027, SAMN14379028, SAMN14379029, and SAMN14379030. The partial and complete gene sequences deposited in GenBank are listed in Table 5. Erythromycin-resistant isolates harboured identical ribosomal mutations and CITCc1303-18 partial sequences were submitted. One representative *gyrA* GT was submitted for each type. 

## 5. Conclusions

Although non-poultry sources contribute to campylobacteriosis incidence, poultry are natural thermophilic *Campylobacter* spp. hosts. The broiler industry serves as a reservoir for the dissemination of resistant campylobacters. The enrichment and stability of *Campylobacter* spp. resistance determinants is noteworthy but the natural competence and potential of recombination or acquisition of mobile genetic elements contributes to the *Campylobacter*. Taken together, the data collected in this study point to the diversity of resistance determinants circulating among Irish broilers, contributing to the development of resistance to clinically relevant antimicrobials. 

## Figures and Tables

**Table 1:** GyrA sequence types (GTs) and associated polymorphisms distributed among 85 ciprofloxacin-resistant *C. jejuni* isolates (GTJs) and 14 ciprofloxacin-resistant *C. coli* isolates (GTCs). Polymorphisms causing an amino acid substitution (nonsynonymous mutations) are highlighted in black.

**GTJ**	***n*** **= (%)**	**Nucleotide Position (Base Indicated in Brackets) of Wild-type Strain *C. jejuni* NCTC 11168 *gyrA* (GenBank Accession Number: L04566.1 and AL111168.1)**										
		**64 (A)**	**72 (T)**	**243 (C)**	**257 (C)**	**357 (T)**	**360 (C)**	**471 (C)**	**483 (T)**	**608 (A)**	**616 (G)**	**622 (T)**
GTJ-I	40 (47.1%)	.	.	.	T	.	.	.	.	.	.	.
GTJ-II	30 (35.3%)	G	C	T	T	C	T	T	C	G	.	T
GTJ-III	15 (17.7%)	.	C	T	T	C	T	T	C	G	A	T
**GTC**	***n*** **= (%)**	**Nucleotide Position (Base Indicated in Brackets) of Wild-type Strain *C. coli* NCTC 11366 *gyrA* (GenBank Accession Number: AF092101.1 and NZ_UIGM01000003.1)**										
		**117 (T)**		**252 (C)**	**257 (C)**		**297 (C)**	**342 (T)**		**471 (T)**	**498 (G)**	
GTC-I	1 (7.1)	.		.	T		C	.		T	.	
GTC-II	7 (50)	C		.	T		C	.		T	.	
GTC-III	1 (7.1)	C		.	T		.	.		T	.	
GTC-IV	1 (7.1)	.		.	T		C	.		.	.	
GTC-V	2 (14.3)	.		T	T		C	.		T	.	
GTC-VI	1 (7.1)	.		.	T		C	.		.	A	
GTC-VII	1 (7.1)	.		.	T		C	C		T	.	

**Table 2:** Percentage identity (percentage cover in brackets) of mosaic tetracycline resistance gene (*tetO/32/O*) type II in three tetracycline-resistant *Campylobacter* spp. isolates detected in this study and *Streptococcus suis tetO/32/O* gene (GenBank accession number: KY994102.1).

Species/Strain	*C. jejuni* CITCj625-18 *tetO/32/O*	*C. jejuni* CITC727-18 *tetO/32/O*	*C. coli* CITCc-3448-18 *tetO/32/O*	KY994102.1 *S. suistetO/32/O*
***C. jejuni* CITCj625-18 *tetO/32/O***	100% (100%)	100% (100%)	99.78% (96%)	99.73% (96%)
***C. jejuni* CITC727-18 *tetO/32/O***	100% (100%)	100% (100%)	99.78% (96%)	99.73% (96%)
***C. coli* CITCc-3448-18 *tetO/32/O***	99.78% (100%)	99.78% (100%)	100% (100%)	99.95% (100%)
**KY994102.1 *S. suis tetO/32/O***	99.73% (100%)	99.73% (100%)	99.95% (96%)	100% (100%)

**Table 3:** Description of CITCj625-18 (representative of CITCj727-18) multidrug resistance genomic island.

Locus Tag	Gene Annotation	InterProScan Protein Family or Domain	GenBank Accession Number of Closest (% Identity/% Coverage)	Predicted Function
HBF06_00624	Holliday junction ATP-dependent DNA helicase, RuvB	IPR004605	WP_002856258.1 100/100	Holliday junction helicase (strand exchange reactions in homologous recombination)
HBF06_00625	Histidine phosphotransferase	IPR036641	MPA99107.1 100/90)	Signal transduction
HBF06_00627	Autotransporter adhesion, CapC	IPR005546	EAK6247206.1 99.16/94	Protein secretion
HBF06_00628	Truncated tetracycline resistance ribosomal protection protein, TetO	IPR035650	AUA17601.1 99.59/82	Ribosomal protection protein conferring tetracycline resistance (TetO)
HBF06_00629	Aminoglycoside 6- nucleotidyltransferase, ANT(6)-Ib	IPR007530	WP_001255868.1 100/100	Adenylyltransferase activity conferring resistance to aminoglycosides
HBF06_00630	Replication protein, RepB	IPR002631	WP_052777339.1 100/100	Plasmid replication protein
HBF06_00631	DNA topoisomerase	IPR000380	WP_139898553.1 99.52/100	Topoisomerisation and single stranded breakage during transcription, DNA replication and recombination.
HBF06_00632	Streptothricin acetyltransferase, SatA	IPR008125	EOO12820.1 96.86/100	Acetylation of streptothricin conferring resistance
HBF06_00633	Mosaic tetracycline resistance ribosomal protection protein, TetO/32/O	IPR035650	WP_052855148.1 100/100	Ribosomal protection protein conferring tetracycline resistance
HBF06_00634	Plasmid replication protein	None detected	EDP4862066.1 99.21/97	Plasmid replication

**Table 4:** Primer sequences and target genes used for the detection of antimicrobial resistance determinants in resistant thermophilic *Campylobacter* spp. isolates and sialylated lipooligosaccharide (LOS) locus classes A, B and C in streptomycin-resistant *C. jejuni* isolates.

Primer	Type	Sequence (5’ - 3’)	Target	Amplicon Size (bp)	Annealing (°C )	Reference
GZgyrA5	A + S	ATT TTT AGC AAA GAT TCT GAT	QRDR region of *gyrA*	673	50	[15]
GZgyrA6		CCA TAA ATT ATT CCA CCT GT				
TetO-FW	A	ACG GAR AGT TTA TTG TAT ACC	*tetO*	171	52	[103]
TetO-RV		TGG CGT ATC TAT AAT GTT GAC				
TetO/W/O-F	A	ATC CAG ACA GCA GTG ACA TC	*tetO/W/O*	489	50	This study
TetO/W/O-R		ATG ATA GAC CGG AAA CAG GG				
TetO/32/O-i-F	A	GAT ACA ATG AAT TTG GAG CG	*tetO/32/O* type I	545	48	This study
TetO/32/O-i-R		AAT TGT CTT TTG CAC TCC C				
TetO/32/O-ii-F	A	CGG GCA GGT TTT TAAG ATT G	*tetO/32/Oi* type II	365	50	This study
TetO/32/O-ii-R		CTG TAT CAG CAA TCT CTG CG				
F2-campy-23S	A + S	AAT TGA TGG GGT TAG CAT TAG C	Domain V of 23S rRNA	316	55	[121]
R2-campy-23S		CAA CAA TGG CTC ATA TAC AAC TTG				
L4C-F	A + S	TTA TCC CTC TTT TGT AAT AGA TTC TAA	*rplD*	614	48	[61]
L4C-R		ATG AGT AAA GTA GTT GTT TTA AAT GAT				
L22C-F	A + S	TTA GCT TTC CTT TTT CAC TGT TGC TTT	*rplV*	425	48	[61]
L22C-R		ATG AGT AAA GCA TTA ATT AAA TTC ATA AG				
erm(B)-F	A	GGG CAT TTA ACG ACG AAA CTG G	*ermB*	421	52	[111]
erm(B)-R		CTG TGG TAT GGC GGG TAA GT				
CmecoliF3	A + S	AATGTTTTAGCCGATACT	*cmeABC*	428	45	[28]
CmecoliR4		AACACCGCTTACTTGAGG				
cst-II-F	A	ATG AAA AAA GTT ATT ATT GCT GGA AAT G	LOS locus class A/B	885/876	50	[122,123]
cst-II-R		TTA TTT TCC TTT GAA ATA ATG CTT TAT				
orf14c-F	A	CAA CTT TGC AAA ATG ATT TTA TCT ATC ATT	LOS locus class C	995	50	[123]
orf14c-R		ATG CAA ATA CAA CAA AAC AAT TC				

A, amplification; A + S, amplification and sequencing

**Table 5:** Accession numbers of partial sequences submitted to GenBank in this study.

Isolate	Gene	GenBank Accession Number	Sequence Length (bp)
*C. coli* (CITCc1303-18)	23S ribosomal RNA, partial sequence	MT155934	262
*C. coli* (CITCc1303-18)	50S ribosomal protein L4 (*rplD*) gene, partial cds	MT155935	519
*C. coli* (CITCc1303-18)	50S ribosomal protein L22 (*rplV*) gene, partial cds	MT155936	319
*C. jejuni*( CITCj4193-17)	*gyrA* (GTJ-I), partial cds	MT176407	644
*C. jejuni* (CITCj999-18)	*gyrA* (GTJ-II), partial cds	MT176408	644
*C. jejuni* (CITCj193-18)	*gyrA* (GTJ-III), partial cds	MT176409	644
*C. coli* (CITCc3796-B-18)	*gyrA* (GTC-I), partial cds	MT176400	550
*C. coli* (CITCc3636-B-17	*gyrA* (GTC-II), partial cds	MT176401	550
*C. coli* (CITCc3521-18)	*gyrA* (GTC-III), partial cds	MT176402	550
*C. coli* (CITCc3318-17)	*gyrA* (GTC-IV), partial cds	MT176403	550
*C. coli* (CITCc1631-18)	*gyrA* (GTC-V), partial cds	MT176404	550
*C. coli* (CITCc3790-18)	*gyrA* (GTC-VI), partial cds	MT176405	550
*C. coli* (CITCc1303-18)	*gyrA* (GTC-VII), partial cds	MT176406	550
*C. jejuni* (CITCj625-18)	*tetO/32/O* type II, complete cds	MT176410	1920
*C. jejuni* (CITcj727-18)	*tetO/32/O* type II, complete cds	MT176411	1920
*C. coli* (CITCc3448-18)	*tetO/32/O* type II, complete cds	MT176412	1920
*C. jejuni* (CITCj625-18)	*ant(6)-Ib*, complete cds	MT176413	867
*C. jejuni* (CITcj727-18)	*ant(6)-Ib*, complete cds	MT176414	867
*C. coli* (CITCc3448-18)	*ant(6)-Ie*, complete cds	MT176415	900
*C. coli* (CITCc1631-18)	*ant(6)-Ie*, complete cds	MT176416	550
